# Outcome of Pantalar Fusion With Femoral Head Allograft in Avascular Necrosis of Talus

**DOI:** 10.3389/fsurg.2021.658788

**Published:** 2021-09-30

**Authors:** Thiru Karthikeyan Ramu, Mohd Yazid Bajuri, Muhammad Fathi Hayyun, Norliyana Mazli

**Affiliations:** Department of Orthopedics and Traumatology, Universiti Kebangsaan Malaysia Medical Centre, Hospital Canselor Tuanku Mukhriz, Kuala Lumpur, Malaysia

**Keywords:** avascular necrosis, talus, hindfoot arthrodesis nail, femoral head, allograft

## Abstract

**Background:** Avascular necrosis (AVN) of the talus is a challenging condition that is caused primarily by trauma. The severity of the talus fracture determines the risk of AVN. Severe osteonecrosis with the loss of talar integrity can be treated with arthrodesis and structural bone graft.

**Method:** This study shows the experience of pantalar arthrodesis using hindfoot arthrodesis nail, screw fixation, and femoral head allograft in four patients.

**Result:** All patients were satisfied in terms of pain and function after an average of 4 months postsurgery. Limb length discrepancy was <1 cm and hindfoot fusion was achieved by 3 months. The mean score for SF-36 physical function and AOFAS hindfoot score at a 2-year postpantalar arthrodesis was 88 and 80.8, respectively.

**Conclusion:** Hindfoot ankle arthrodesis, with the usage of femoral head allograft, can be successfully used for the treatment of traumatic AVN of talus.

## Introduction

Avascular necrosis (AVN) of the talus can be a challenging condition to manage, and it causes significant disability to the patient (1). It is commonly caused by a fracture of the talus. Atraumatic causes of AVN of the talus include chronic alcoholism, dyslipidemia, steroid use, and idiopathic cause (2). The severity of trauma to the talus increases the risk of AVN and needs to be anticipated in displaced fracture of the talus (3).

Treatment modalities to treat AVN of the talus can be divided into conservative management and surgical management. Conservative measures include non-weight bearing or protected weight bearing with splints and the usage of extracorporeal shock wave therapy. Surgical procedures can be classified into joint sparing procedures (core decompression or bone grafting), joint sacrificing procedures (partial or total ankle replacement), and joint salvage procedures (arthrodesis of ankle or talectomy). Techniques that can be considered for ankle fusion in AVN talus include tibiotalar arthrodesis with screw fixation, tibiotalocalcaneal arthrodesis, and tibiocalcaneal arthrodesis (4). When considering fixation, it is important to understand the stability of the fixation to prevent the danger of implant failure (5).

This study highlights the outcome of pantalar arthrodesis using hindfoot arthrodesis nail, screw fixation, and femoral head allograft in cases of AVN of talus.

## Patients and Methods

This is a single center study involving four patients who underwent pantalar arthrodesis between January 2016 and January 2018. The medical board has approved the study. Verbal and written consent was obtained from the patient regarding the procedure and for the data to be used in this study. There were three males and one female patient. All patients had AVN of the talus secondary to trauma. The patients were not obese and of a medium build. The patients did not have any medical illnesses such as diabetes and osteoporosis.

Patient 1 is a 56-year-old male with a history of falling from the staircase with his ankle in an inverted position. This patient was initially treated conservatively; however, he complained of pain after 6 months, and during follow-up, the talus showed AVN of the talus with the collapse of talus.

**Figure 1:**
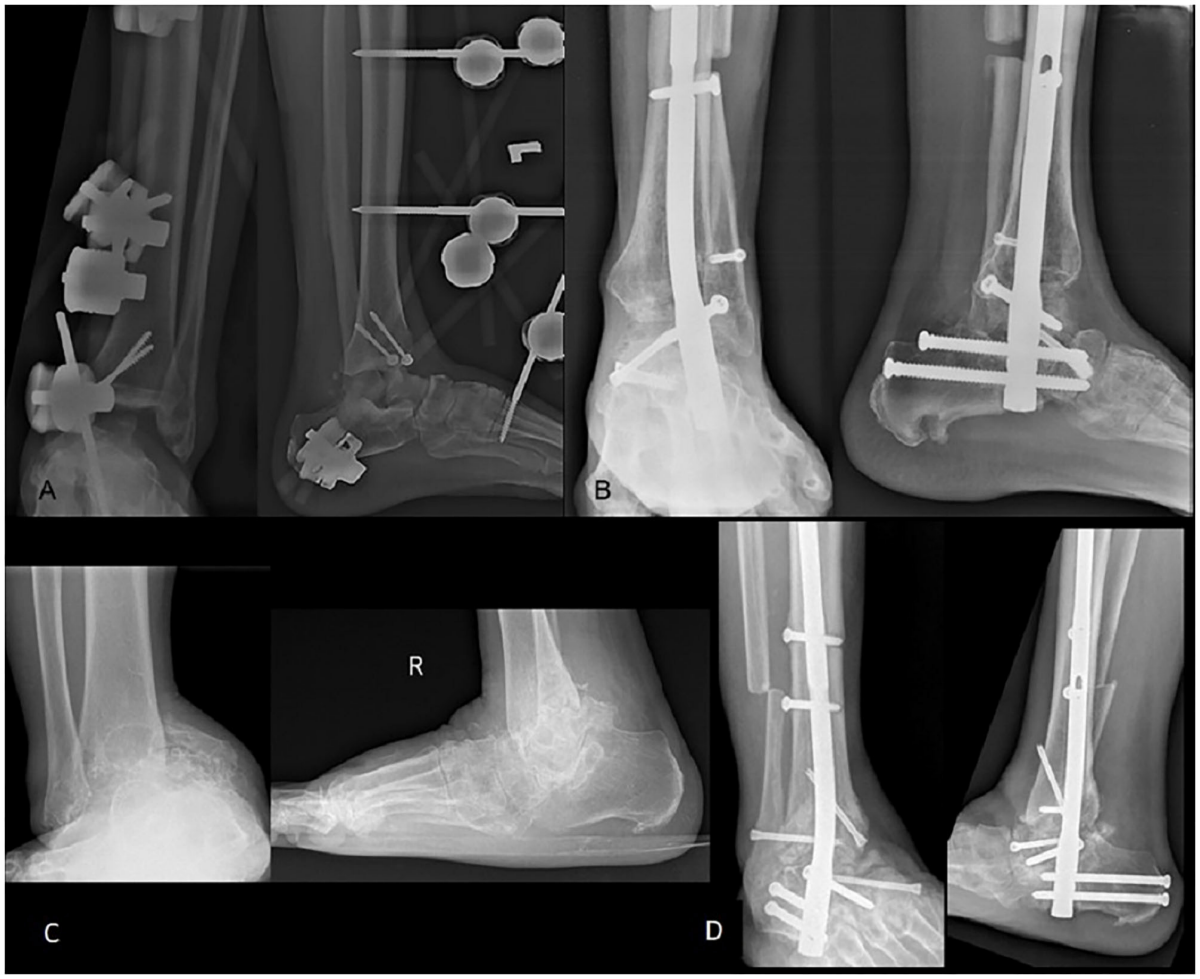
Radiograph of left tibia and fibula post ankle external fixator **(A)** and 1 year post ankle arthrodesis **(B)**. Radiograph showing the destruction of the talus with ankle deformity **(C)**. Ankle alignment was restored post ankle arthrodesis with femoral head allograft **(D)**.

Patient 2 is a 48-year-old male who sustained an open fracture of the left medial malleolus, a comminuted fracture of the left talus, and a closed fracture of the calcaneum after a motor vehicle accident (MVA) in early 2016. The initial surgery was wound debridement of the left ankle, screw fixation of the medial malleolus, and cross ankle external fixation followed by ankle arthrodesis a few months later (Figure 1A). After the wound healed, the patient was referred for further management in view of AVN of the talus.

Patient 3 is a 68-year-old male with a history of a twisted ankle while walking after tripping in a pothole. The patient initially did not seek any treatment, but after 4 years of injury came with pain over the right ankle. The radiograph showed AVN of the right talus with subluxated ankle joint (Figure 1C).

Patient 4 is a 23-year-old lady who was involved in MVA in 2016 and sustained closed fracture of the right talus. Patient was initially treated conservatively. It was complicated with AVN of the talus at 3 months follow-up.

Upon clinical examination, the range of motion for all the patients is between 0° and 5° of plantar flexion with tenderness around the ankle and subtalar joint.

Tibiotalocalcaneal arthrodesis was performed using the Synthes® Expert Hindfoot Arthrodesis Nail with additional femoral head allograft were used to fill the void due to severe destruction of the talus. All cases were operated by a single foot and ankle surgeon using a similar technique. Patients were assessed at an interval of 2 weeks, 6 weeks, 3 months, 6 months, 1 year, and 2 years postoperatively in the clinic. Non-weight bearing ambulation was advocated for the first 6 weeks and then followed by protected weight-bearing with crutches for another 6 weeks. Patients were allowed to bear weight fully after 3 months.

The patients were assessed both clinically and radiologically. Assessment was done using the validated American Orthopedic Foot and Ankle Society (AOFAS) hindfoot score and SF-36 score. Scores for the AOFAS scale are divided into points for pain, functional, and alignment with a total of 100 points. SF-36 scoring consists of thirty-six questions to assess the general well-being and functional status of the patient. Union was defined as stable hindfoot clinically with preserved function to ambulate with or without braces and evidence of radiological union.

## Operative Technique

All patients underwent hindfoot arthrodesis using the Synthes® Expert Hindfoot Arthrodesis Nail. Patients were positioned supine on the operating table. An inverted J incision was started from the tip of the lateral malleolus which extend to 10 cm above the lateral malleolus and the incision extended distally toward the fourth metatarsal. Fibulectomy was done at 10 cm from the tip of the lateral malleolus. A periosteal elevator was used to strip the fibular periosteum and expose the distal tibia, tibiotalar joint, posterior facet of the subtalar joint, and sinus tarsi. Another curve incision was made from the medial malleolus and it extended distally toward the talonavicular joint. The ankle capsules, medial subtalar, and talonavicular joint were exposed and denudation of the cartilage was done.

**Figure 2:**
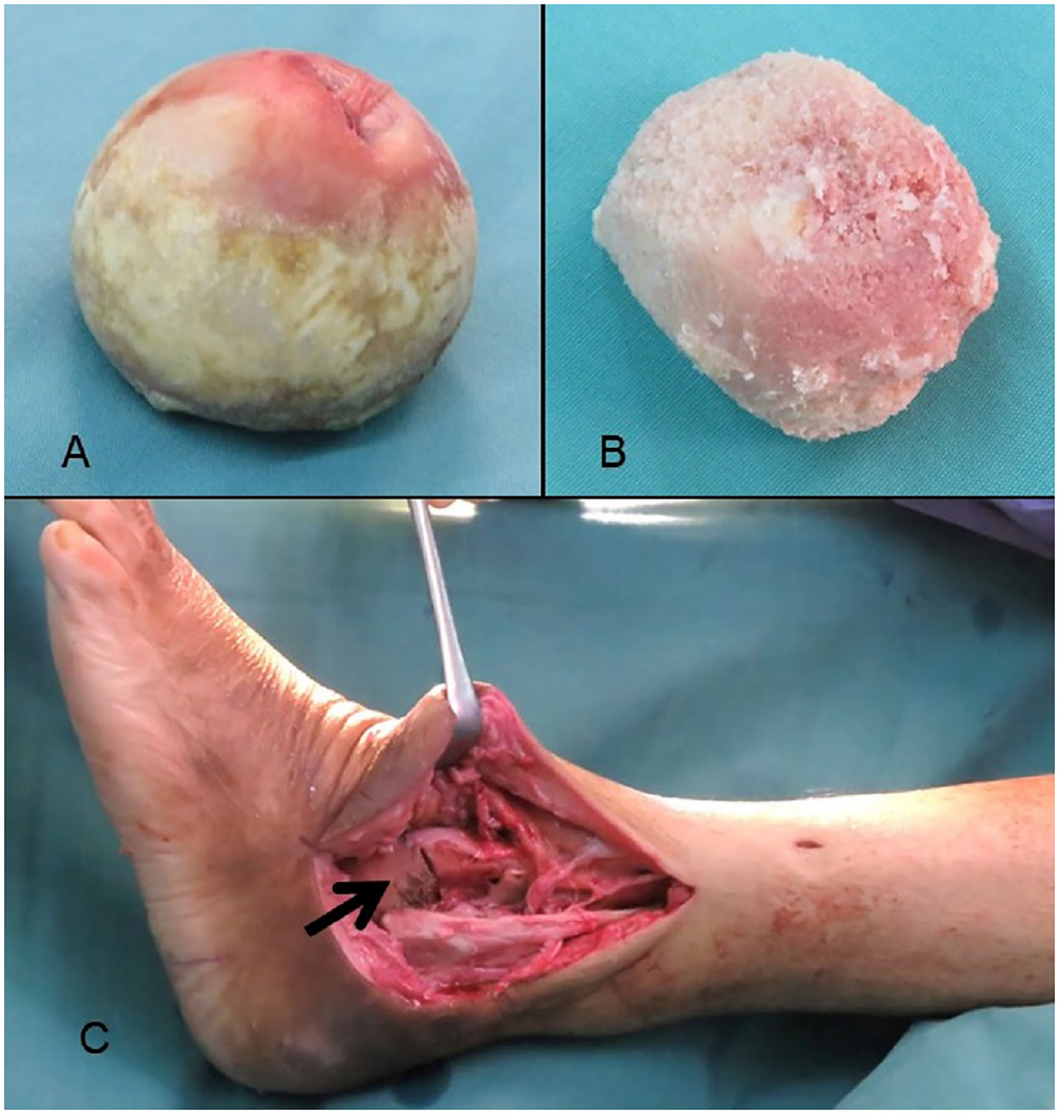
Femoral head allograft before **(A)** and after removal of cartilage **(B)**. Femoral head allograft was inserted to replace the talar bone loss **(C)**.

Once the joints are prepared and proper denudation of the cartilage has been achieved, we prepared the femoral head allograft. These are frozen femoral head allografts from the bone bank and the femoral head was thawed in normal saline for 45 min before removing the cartilage over the femoral head (Figure 2). Removal of the cartilage is important for better graft incorporation into the recipient bones. The allograft was shaped according to the size of the bone gap that needs to be filled at the ankle joint. Before insertion, the allograft was washed with hydrogen peroxide, povidone iodine, gentamycin, and sterile water copiously. The allograft was inserted and stabilized with K-wires. The placement of the graft and the foot alignment was checked with an image intensifier. Hindfoot alignment of 5° valgus, 5° external rotation, and plantigrade position were achieved. Entry site is in line with the tibial canal and the lateral column of the calcaneus. By using an image intensifier, the center of the tibial canal is identified and a line is drawn. The center of the lateral column of the calcaneus is palpated and another line is drawn. The entry point is located at the intersection of these two lines and should be in line with the longitudinal axis of the foot. Hindfoot nail was inserted and the interlocking screws were secured. Final reduction and stabilization were checked with the image intensifier. The talonavicular fusion was done using screw fixation.

## Results

All patients expressed satisfaction in terms of pain and function after an average of 4 months postsurgery and were able to ambulate independently without any support. Radiological assessment of the ankles showed that all patients achieved union (Figures 1B,D) at 14–16 weeks postsurgery. Limb length discrepancy was <1 cm for all patients, and there was no compensatory pelvic tilt or short leg gait during ambulation. There was no surgical site infection seen in all the patients.

**Figure 3:**
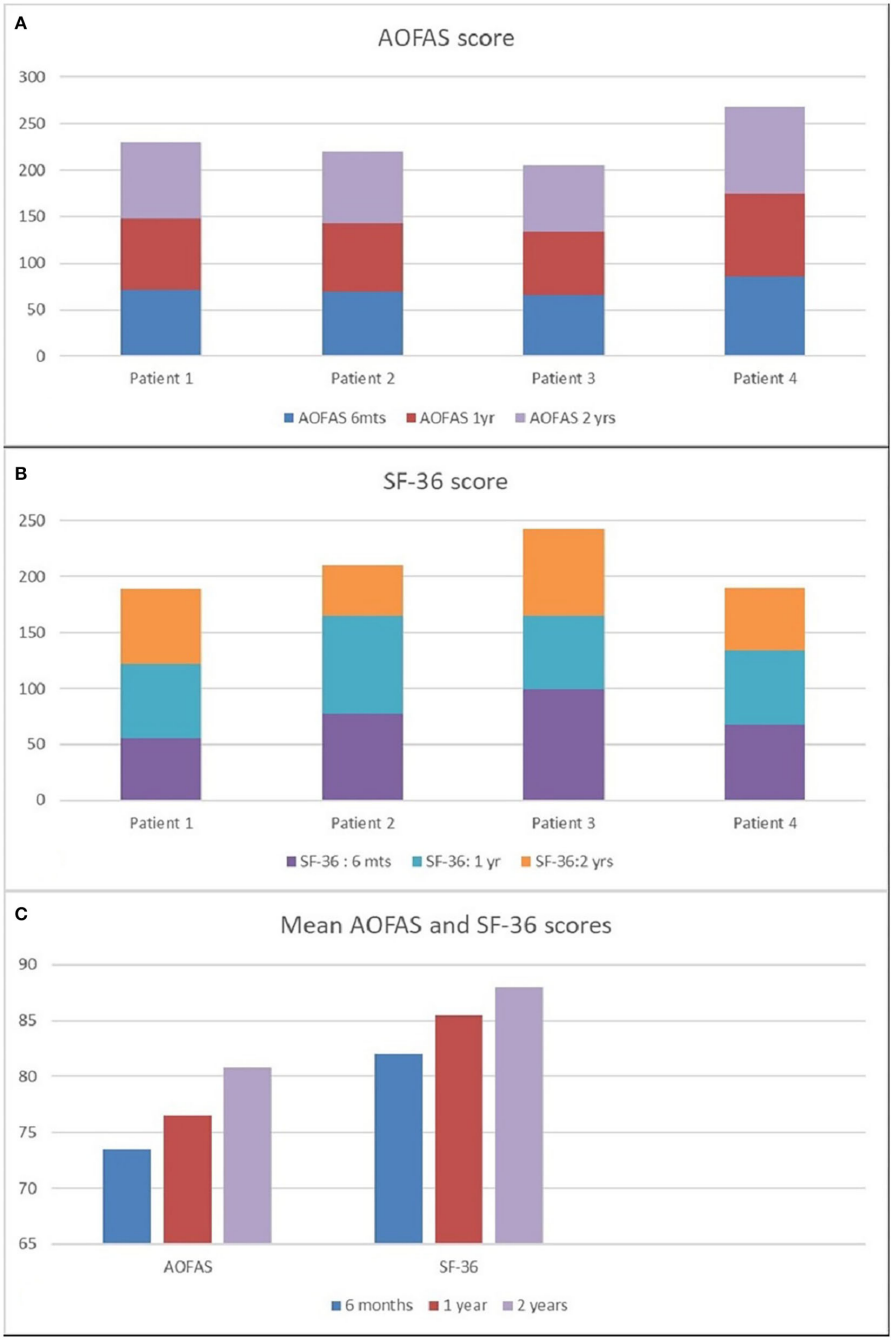
The AOFAS hindfoot score at 6 months, 1 year, and 2 years postoperatively **(A)**. SF-36 score 6 months, 1 year, and 2 years postoperatively **(B)**. Mean AOFAS score and SF-36 score at 6 months, 1 year and 2 years postoperatively **(C)**.

The American Orthopedic Foot and Ankle Society hindfoot score and SF-36 were scored for all the patients at 6 months, 1 year, and 2 years postoperatively, and are tabulated as shown in Table 1 and Figures 3A,B. The mean score for SF-36 physical function and AOFAS hindfoot score was 82 and 73.5, respectively, at 6 months, 85.5 and 76.5, respectively, at 1 year, 88 and 80.8 at 2 years postoperatively, as shown in Figure 3C.

**Table 1:** AOFAS and SF-36 scores 6 months, 1 year, and 2 years postoperatively.

	**AOFAS scale**			**SF-36 (physical functioning)**		
	**6 months**	**1 year**	**2 years**	**6 months**	**1 year**	**2 years**
Patient 1	72	76	82	80	84	87
Patient 2	70	73	77	78	82	83
Patient 3	66	68	71	84	87	90
Patient 4	86	89	93	86	89	92

## Discussion

Avascular necrosis of the talus can be managed conservatively or with surgical intervention. Conservative measures include non-weight bearing or protected weight bearing with splints and the usage of extracorporeal shock wave therapy (4). Extracorporeal shock wave therapy has been shown to improve the AOFAS hindfoot score significantly compared with physical therapy. The surgical intervention can be delayed if the patient responds to conservative treatment as no study has shown the association of delay in surgery with worse outcome. A recent study suggests that usage of bisphosphonate in the early stage of talar AVN may delay the development of arthritis (6).

Surgical procedures can be classified into joint-sparing procedures (core decompression or bone grafting), joint sacrificing procedures (partial or total ankle replacement), and joint salvage procedures (arthrodesis of ankle or talectomy) (4). The severity of osteonecrosis of the talus and the presence of arthritis changes are important factors in determining the type of surgery. The degree of AVN by Ficat and Arlet is determined from a plain radiograph of the talus. The joint-sparing procedures are suitable for grade I–III but not when the ankle joint has developed arthritis changes (grade IV).

The pantalar arthrodesis in this series acts as a salvage operation for patients with AVN of the talus with ankle pain. The aim of the surgery is to improve limb function and quality of life. All patients have undergone hindfoot arthrodesis using the hindfoot nail with dried frozen femoral head allograft. The function of the allograft is to fill the void after the removal of the non-viable talus and to maintain the limb length postoperatively. Other conditions which can lead to large defects which need allograft are previous pilon or talus fracture, failed total ankle replacement surgery, charcot arthropathy, and osteomyelitis. The femoral head allograft was chosen as the graft of choice as it can fill up a large gap in the joint, maintain adequate limb length, and provide structural support for the fused joint (7). The shortened limb can lead to back pain, knee and hip osteoarthritis, and pelvic tilt as compensatory mechanisms (8).

Among the challenges of ankle arthrodesis with large bone block allograft is the incorporation of the graft into host bone and union. We have made multiple small holes on the bony interface of the graft and the bone using a 2.5-mm drill to increase the chances of bone incorporation of the graft. It was reported that ankle arthrodesis without a large allograft showed higher rates of union of 86–100%, with shortening of the limb (8).

The presence of diabetes mellitus has been a significant factor that led to non-union in pantalar arthrodesis. Jeng et al. reported that among their 32 patients who underwent bone block ankle arthrodesis, all nine patients with diabetes mellitus did not achieve radiological union (7). However, these patients were able to ambulate with or without a brace, and they were classified as stable non-union and did not require further surgery. Two patients in this study have insulin-dependent diabetes mellitus, which was controlled, and have achieved radiological and clinical union. Another study also compared the union rates with several intraoperative factors such as surgical approach, preparation technique of host bone, and difference between intramedullary rod and plate. It was reported that none of these factors affected the union rates (9).

Several authors have reported tibiotalocalcaneal arthrodesis with autograft augmentation and direct apposition without graft. Culpan et al. presented a study of 16 patients who underwent tibiotalocalcaneal arthrodesis with tricortical iliac crest autograft for failed total ankle arthroplasty, which showed 93% fusion rates (6). Another study reported bony union in 66.7% of the cases following resection of necrotic talar body and tibiotalocalcaneal fusion with bulk autograft from the posterior iliac crest (10). However, autograft may not provide the required graft volume to fill the bone void, and autograft harvesting has its complications of donor site morbidity such as pain and infection.

Hopgood et al. analyzed the outcome of ankle fusion with direct apposition of the bones without the usage of bone graft in the presence of a large bone gap. Fusion was achieved in 74% of patients, but with the complication of limb shortening (8). Another study reported union in four cases of AVN involving the entire body of the talus. The remaining talar head–neck portion was fused to the anterior aspect of the tibia following the tibiocalcaneal arthrodesis (9). The usage of bone substitutes and cement, such as using intramedullary cement osteosynthesis, is able to augment the treatment of bone fracture (11).

Jeng et al. reported an SF-12 score of 35 for physical component and 57 for mental component for patients who underwent hindfoot arthrodesis with bulk femoral head allograft (7). The mean SF-36 score in our series is 88, and the score correlates with the clinical improvements of the patient. The AOFAS score in our case series showed good results and all of them were satisfied with the outcome of the hindfoot arthrodesis. One patient had moderate pain over the ankle whereas the other three patients had very mild pain during daily activities. Adrian et al. also showed good to excellent AOFAS scores post ankle arthrodesis in a study of 16 patients who underwent ankle fusion for AVN of the talus (7).

All patients in this study achieved successful fusion by 14–16 weeks. Therefore, pantalar arthrodesis with the usage of femoral head allograft can be successfully used for the treatment of traumatic AVN of talus.

## Data Availability Statement

The original contributions presented in the study are included in the article/supplementary material, further inquiries can be directed to the corresponding author/s.

## Ethics Statement

Ethical review and approval was not required for the study on human participants in accordance with the local legislation and institutional requirements. The patients/participants provided their written informed consent to participate in this study. Written informed consent was obtained from the individual(s) for the publication of any potentially identifiable images or data included in this article.

## Author Contributions

TR, MH, MB, and NM: writing manuscript, collecting data, analyzing the data, and proofreading. All authors contributed to the article and approved the submitted version.

## Conflict of Interest

The authors declare that the research was conducted in the absence of any commercial or financial relationships that could be construed as a potential conflict of interest.

## Publisher's Note

All claims expressed in this article are solely those of the authors and do not necessarily represent those of their affiliated organizations, or those of the publisher, the editors and the reviewers. Any product that may be evaluated in this article, or claim that may be made by its manufacturer, is not guaranteed or endorsed by the publisher.
